# Defining minimum media requirements for *ex vivo* respiratory cilia studies using mouse tracheal tissue

**DOI:** 10.7717/peerj.21674

**Published:** 2026-09-07

**Authors:** Richard Francis

**Affiliations:** Cilia Research Laboratory, College of Medicine and Dentistry, James Cook University, Townsville, Queensland, Australia

**Keywords:** Cilia, Respiratory epithelium, Cilia beat frequency, Cilia generated flow

## Abstract

Freshly harvested airway samples are routinely collected and imaged to study cilia function and diagnose motile ciliopathies. A previous study has suggested that Dulbecco’s phosphate-buffered saline (DPBS) is just as effective as complex traditional cell culture media for the *ex vivo* assessment of cilia motility using mouse airway samples. The use of a less complex media to study cilia motility would be beneficial in reducing exogenous factors typically found in complex cell culture media that may influence cilia behaviour. Thus, the aim of this study was to determine the minimum media composition required to preserve airway ciliary function by incubating isolated mouse trachea sections in L15 and three simple saline-based solutions of increasing complexity (0.9% Saline, NaCl/KCl solution containing 150 mM NaCl/4.15 mM KCl, DPBS) following 0–12 hour storage at either 4 °C or 37.5 °C. Results reveal that a simple NaCl/KCl solution which approximates physiological extracellular concentrations of Na^+^, Cl^−^, and K^+^ was sufficient to preserve ciliary motility in these tissues for up to 2 hours at 37.5 °C. Beyond 2 hours, samples stored in all solutions lacking an exogenous energy source displayed small but significant reductions in cilia beat frequency (CBF), which could be prevented by 10 mM glucose supplementation, maintaining CBF for up to 12 hours. In contrast, storage in 0.9% saline led to a rapid and significant impairment of cilia motility, with this impairment being significantly more pronounced following 4 °C storage *vs*. 37.5 °C storage. Additionally, ciliated cells were found to be more susceptible to storage associated injury and cell death than non-ciliated epithelial cells, with this vulnerability exacerbated by storage in 0.9% saline and at 4 °C. In conclusion, a simple NaCl/KCl solution is sufficient to maintain ciliary motility for up to 2 hours at 37.5 °C, with glucose supplementation alone required to preserve CBF for up to 12 hours. The use of simplified media for cilia motility studies provides a standardized and cost-effective foundation for future mechanistic studies allowing clearer insights into underlying pathways regulating motile cilia function.

## Introduction

Cilia are hairlike structures lining the respiratory epithelial surface and beat in a coordinated manner to remove mucus and inhaled foreign particles from the lungs in a process called mucociliary clearance (Bustamante-Marin & Ostrowski, 2017; Fahy & Dickey, 2010). Disruption of normal mucociliary clearance contributes to a range of respiratory diseases, including chronic obstructive pulmonary disease (COPD), asthma, cystic fibrosis, and primary ciliary dyskinesia (Tilley et al., 2015; Wallmeier et al., 2020). Understanding the mechanisms that regulate normal ciliary motility is essential for elucidating both normal respiratory physiology and the pathophysiology of human disease. To better characterise the fundamental regulation of ciliary function, reducing exogenous factors present in complex cell culture media may be beneficial, as these components could independently influence ciliary behaviour.

Due to the difficulty in directly imaging and experimentally manipulating respiratory cilia, a variety of *ex vivo* approaches have been developed to study their function. These can be divided into acute assessment models using freshly harvested airway tissue (Francis, 2025b; Lever et al., 2024), or primary cell culture models using respiratory epithelia cells grown over multiple weeks after they have formed ciliated spheroids (Marthin et al., 2017; van der Vaart et al., 2021) or ciliated cell sheets (Liu et al., 2021; Yasuda et al., 2020) depending on culture methods used. These *ex vivo* studies rely on a diverse range of storage/imaging conditions and culture media formulations, which introduce a variety of uncontrolled variables making direct comparisons between published studies difficult. To determine whether different media or storage conditions influence motile cilia function, a range of commonly used media for studying respiratory cilia was previously evaluated (Francis, 2025a). These results showed that all commonly used media appeared similarly effective in maintaining motile activity in mouse respiratory cilia for up to 12 h following storage at both 4 °C and room temperature (Francis, 2025a). Unexpectedly, Dulbecco’s phosphate-buffered saline (DPBS) was found to preserve ciliary motility in fresh airway samples just as effectively as more complex, nutrient-rich media (*e.g.*, Roswell Park Memorial Institute medium (RPMI), Dulbecco’s modified eagle’s medium (DMEM), Leibovitz’s L-15 medium (L-15)) (Francis, 2025a). These findings prompted the current study which aims to determine what is the minimal media requirements necessary to maintain respiratory cilia motility *ex vivo*.

Defining the minimal media requirements to study cilia motility is of both practical and conceptual importance. Use of a simpler media to study cilia motility will help minimize the number of exogenous factors found in cell culture media that may influence cilia function, thereby enabling clearer insights into understanding the biology underlying normal ciliary function. Moreover, identifying the most basic solution capable of maintaining motility will provide a standardized and cost-effective foundation for future mechanistic studies. Thus, this study was designed to systematically assess the capacity of three simple saline-based solutions of increasing complexity to preserve motile ciliary function in isolated mouse trachea samples following short-term storage at either 4 °C or 37.5 °C. Surprisingly, it was found that a physiological NaCl/KCl solution was sufficient to maintain normal ciliary activity for up to 2 h following both 4 °C or 37.5 °C storage without measurable impairment, whereas physiological saline alone caused a significant reduction in cilia activity. These results establish important baseline media requirements for *ex vivo* maintenance of respiratory cilia and provide an important foundation for future studies into the molecular and cellular factors regulating mucociliary clearance.

## Materials and Methods

### Animals

All experimental procedures were approved by the James Cook University Animal Ethics Committee (protocol A2783). Adult C57BL/6 mice, scheduled for routine colony culling, were sourced from the Institutional Bioresources Facility. Animals were maintained under controlled environmental conditions in air-conditioned rooms, housed in racked cages with a Smart Flow ventilation system (Tecniplast, Buguggiate, Varese, Italy), and had unrestricted access to standard rodent chow and water with additional environmental enrichment such as bedding and cardboard tubing.

### Airway tissue harvest and sample preparation

Ciliated tracheal epithelial samples were collected for video microscopy as described previously (Francis, 2025b). In brief, mice were euthanized *via* CO_2_ (5 L/min CO_2_ gas using a 9 L euthanasia chamber), and trachea were placed into the media being assessed: Dulbecco’s phosphate buffered saline (DPBS, Sigma-Aldrich; D8662); 0.9% saline (155 mM sodium chloride; UNIVAR, Downers Grove, IL, USA); NaCl/KCl solution containing 150 mM Sodium Chloride + 4.15 mM Potassium Chloride (UNIVAR, Downers Grove, IL, USA); NaCl/KCl + glucose solution containing 150 mM sodium chloride + 4.15 mM potassium chloride + 10 mM Glucose (G7021; Sigma-Aldrich, Burlington, MA, USA); or L-15 media lacking phenol red (21083027; ThermoFisher Scientific, Waltham, MA, USA) supplemented with 10% FBS (16000036; ThermoFisher Scientific, Waltham, MA, USA) and 100 units/mL penicillin G sodium/100 μg/mL streptomycin sulfate (15140122; ThermoFisher Scientific, Waltham, MA, USA).

Trachea lengths were bisected longitudinally through the trachealis muscle and mounted in a shallow imaging chamber (two 24 × 50 mm coverslips separated by a silicone gasket made from 0.254 mm thick silicon sheeting as outlined in Francis (2025b)) with ~100 µL of tested media. A total of 1 µm microspheres (08226-15; Polysciences, Warrington, PA, USA) were added to the media to track cilia-driven flow. Each trachea was divided into four segments, yielding four possible timepoints. One trachea sample was immediately imaged as described below (time = 0 min), other samples were incubated at either 4 °C or 37.5 °C for 30, 60, 120, 360, or 720 min before imaging. Group sizes (*n* = 5 mice) were based off previous studies (Francis, 2025a; Scopulovic et al., 2022). No samples/data were excluded; confounders were not controlled for. 37.5 °C was maintained by keeping samples in a cell culture incubator without CO_2_ (MCO-20AIC; Panasonic, Kadoma, Osaka, Japan), 4 °C was maintained by placing samples in a 4 °C refrigerator (MPR-721, Pharmaceutical Refrigerator; Panasonic, Kadoma, Osaka, Japan). All samples were imaged at 37.5 °C following storage.

### Cilia imaging and motility quantification

Tracheal samples were imaged at 37.5 °C on a Zeiss Axiovert 200 inverted microscope equipped with a heated microscope chamber, differential interference contrast (DIC) filters, and a 63×/1.4 oil-immersion objective. Ciliary motility was recorded using a high-speed USB 3.0 camera (GS3-U3-32S4M-C, GrassHopper3; Teledyne Vision Solutions, Waterloo, ON, Canada) at a resolution of 0.107 µm/pixel (1,440 × 550 field of view). Short recordings (1 second at ~300 fps) were acquired to measure cilia beat frequency (CBF), longer recordings (10 seconds at 30 fps) were acquired to evaluate cilia generated flow. Within each 10 second video, the number of cells with motile cilia was manually counted. CBF was calculated for three randomly chosen ciliated cells (roughly equidistant from each other as per manual assessment) from each 1 second movie using ImageJ (FIJI v2.3.0/1.53q (Schindelin et al., 2012)) together with a custom MATLAB script (https://github.com/ElvPan/CBF-analysis) (Scopulovic et al., 2022). To assess flow, the 1 µm microspheres were tracked within the 10 second movies using the ImageJ Manual Tracking plugin (https://imagej.net/plugins/manual-tracking). Flow velocity was determined from microsphere speed, while flow linearity was quantified as directionality (net displacement/total distance). Microspheres moving along straight paths had directionality values near 1, while microspheres moving with random movement had directionality values approached 0. Microsphere velocity thus provided a measure of flow speed, while directionality indicated the presence of cilia generated linear flow.

### Quantification of cell survival in different media

Cell viability was assessed following 120 or 360 min incubation at either 4 °C or 37.5 °C using live/dead staining, immunohistochemistry, and confocal fluorescence microscopy (as previously described (Francis, 2023)). In brief, samples were stained using the Violet Live/Dead Stain Kit (L34963; ThermoFisher Scientific, Waltham, MA, USA), fixed in 4% paraformaldehyde, then immunolabeled with a mouse monoclonal anti-acetylated tubulin antibody (1:1,000; T7451; Sigma-Aldrich, Burlington, MA, USA) and goat anti-mouse FITC-conjugated IgG antibody (1:500; 115-095-003; Jackson ImmunoResearch, West Grove, PA, USA) with TRITC–phalloidin (1:1,000; P1951; Sigma-Aldrich, Burlington, MA, USA). Samples were mounted on glass slides with Fluoroshield (F6182; Sigma-Aldrich, Burlington, MA, USA) and a coverslip covered with a 0.13 mm silicon sheet gasket to prevent crushing. Slides were then sealed with quick-drying nail polish and stored at 4 °C until imaged.

Stained samples were imaged using a Zeiss LSM 710 confocal microscope, 40x objective (Zeiss EC Plan-Neofluar 40×/1.30 Oil DIC M27), and ZEN Black software (v2.31 SP1). The following excitation/emission settings were used. Blue live/dead staining: 405 nm excitation, 494–552 nm emission. FITC: 488 nm excitation, 494–552 nm emission. TRITC: 561 nm excitation, 566–669 nm emission. Z-stacks were captured at a resolution of 0.21 µm (xy) and 0.4–0.5 µm (z) using a 1,024 × 1,024 pixel field of view with ~30 z-slices. Image analysis was carried out using ImageJ (FIJI v2.3.0/1.53f) (Schindelin et al., 2012). Z-stacks were first converted into maximum intensity projections and merged into composite three-color images. Quantification was performed using the ImageJ ‘Multi-point’ tool to count total epithelial cells (TRITC), ciliated cells (FITC), dead cells (live/dead dye, acetylated tubulin-negative), and dead ciliated cells (live/dead dye, acetylated tubulin-positive).

### Data analysis and statistics

Multiple measurements (≥3) of each parameter (motile cilia abundance, CBF, flow velocity, flow directionality) were made in each tissue sample, which were then averaged for each sample; resultant group averages (samples from *n* = 5 mice per group). *n* = 5 represents biological replicates (individual animals) with technical replicates averaged per sample which were then analysed using two-way analysis of variance (ANOVA) and *post-hoc* Tukey’s multiple comparisons test (Prism 10.6.1; GraphPad Software, San Diego, California, USA). *P* < 0.05 was considered significant.

Author declares no potential conflicts of interest. No grant funding was used to conduct this study. No protocol (research question, key design features, or analysis plan) was prepared for this study. Samples were not analysed blindly; however, this was not considered to be a confounding variable. Firstly, each trachea was divided into four segments, yielding four timepoints per sample allowing each sample to serve as its own timed control. Secondly, cilia motility was assessed using an automatized MATLAB script minimizing possible observer bias.

## Results

A total of 100 mice yielding 350 trachea samples were used for this study (See Table S1 for group list; Raw Data.xlsx for raw data and *P*-Values). In summary, to determine the minimum media requirements to maintain cilia motility in mouse airway samples, liquid media of increasing complexity (Saline, NaCl/KCl, DPBS, L15+FBS) were assessed for their ability to maintain cilia function over time (0–12 hours), following either 4 °C or 37.5 °C storage.

### Immediate effect of harvest media on ciliary function

**Figure 1:**
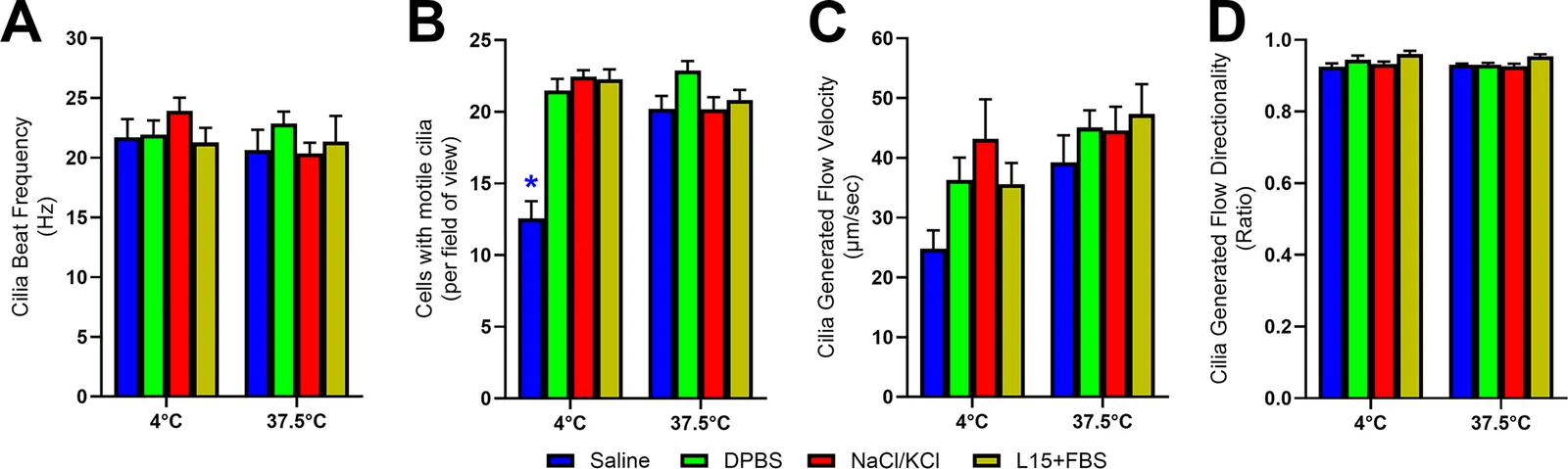
Quantification of tracheal cilia motility immediately following tissue dissection in saline, NaCl/KCl, DPBS, or L-15+FBS at either 4 °C or 37 °C. (A) Cilia beat frequency (Hz). (B) Number of epithelial cells with motile cilia observed per field of view. Cilia-generated flow velocity (C) and flow directionality (D) as determined by tracking microspheres suspended in each medium. Data is presented as mean ± SEM (*n* ≥ 5 mice per media). *P < 0.05 compared with all other cell counts. See Supplemental Material: Raw Data.xlsx for *P*-values.

The immediate effect of saline, DPBS, NaCl/KCl, and L15+FBS on respiratory cilia function was assessed by harvesting/dissecting then immediately imaging tissue within each media (time = 0 min; Fig. 1). No significant difference was seen in CBF measured in any samples harvested at time 0 in any media at either 4 °C or 37.5 °C (Fig. 1A). Observed CBFs ranged between 20.4 ± 2.9 Hz for cells in 4 °C NaCl/KCl up to 23.9 ± 3.6 Hz for cells 37.5 °C NaCl/KCl (Fig. 1A). Motile ciliated cell abundance, as assessed by counting the number of epithelial cells with motile cilia per microscope field of view, showed a significant reduction for samples harvested in 4 °C Saline (12.6 ± 3.6 cells/field) when compared to samples harvested in all other media (Fig. 1B). No significant difference was seen in motile ciliated cell abundance at time 0 within any other media at both 4 °C and 37.5 °C (Fig. 1B), where observed values ranged between 20.2 ± 2.8 cells/field for samples in 37.5 °C NaCl/KCl up to 22.9 ± 2.0 cells/field for samples in 37.5 °C DPBS.

Cilia generated flow was assessed by tracking microsphere movement within the media by quantifying microsphere velocity (Fig. 1C), and microsphere directionality (Fig. 1D). While samples harvested in 4 °C media tended to show a slower flow velocity, with those in 4 °C Saline being noticeably lower, no significant difference was seen in flow velocity for time 0 samples in both 4 °C and 37.5 °C media (Fig. 1C). Cilia generated flow velocity ranged between 24.8 ± 11.7 µm/sec for samples in 4 °C Saline up to 47.3 ± 19.5 µm/sec for samples in 37.5 °C L15+FBS.

All samples displayed strong linear flow across the surface of the ciliated respiratory epithelia as assessed by microsphere directionality (Fig. 1D), with no significant difference found in flow directionality at time = 0 min for samples in any media at both 4 °C and 37.5 °C. All samples in all media, at both 4 °C and 37.5 °C, showed similar high directionality values (>0.9) corresponding to the generation of strong linear flow across all ciliated epithelia measured, with directionality ranging between 0.93 ± 0.04 for samples in 4 °C Saline up to 0.96 ± 0.04 for samples in 4 °C L15+FBS (Fig. 1D).

### Effect of storage media on cilia function following 4 °C storage

**Figure 2:**
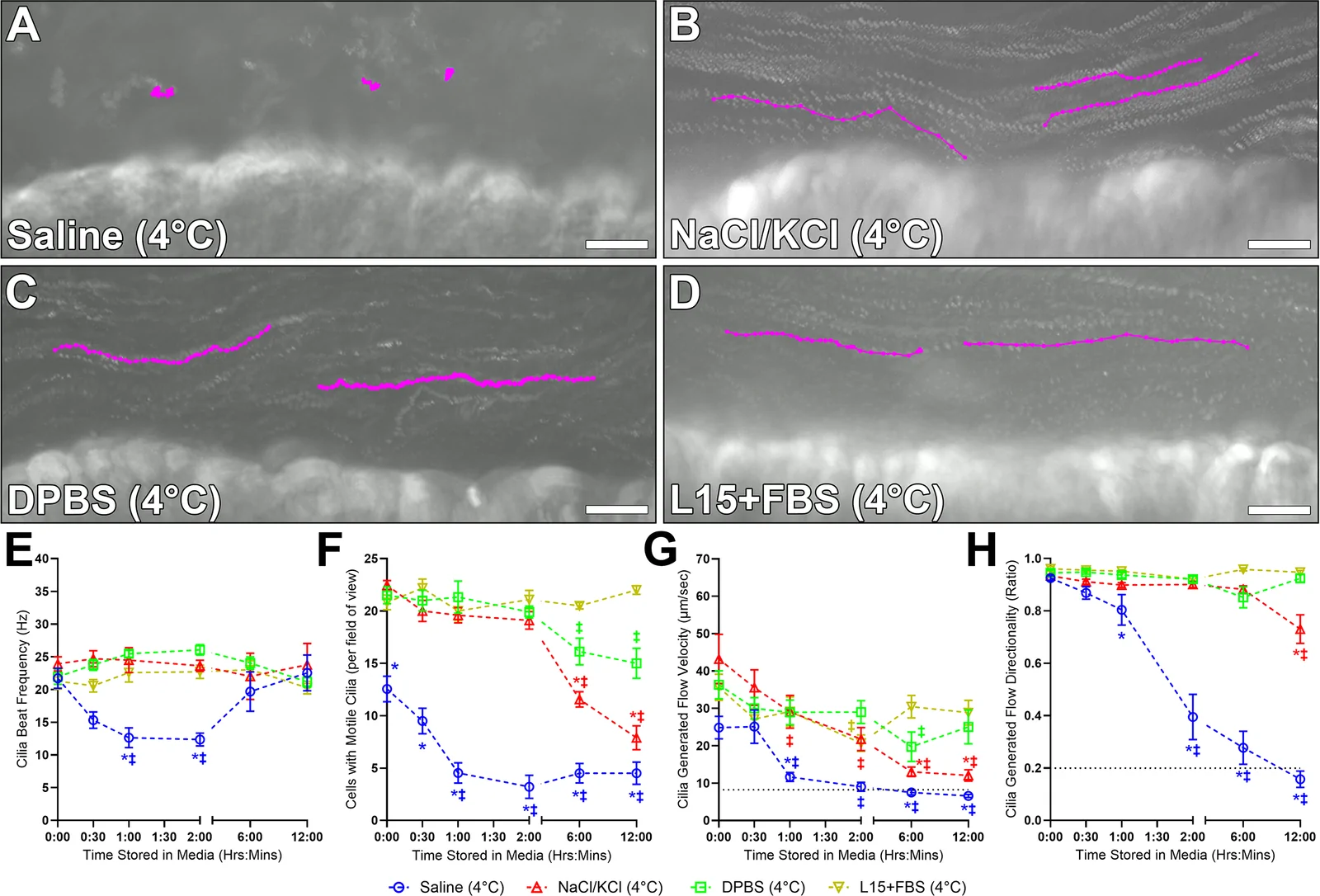
Quantification of cilia motility and cilia-generated flow in tracheal samples following storage at 4 °C for 0, 30, 60, 120, 360, or 720 min. (A–D) Representative Z-projections of 10 s brightfield movies illustrating microsphere movement after 2 h of 4 °C storage in (A) Saline, (B) NaCl/KCl, (C) DPBS, and (D) L15+FBS. Purple traces indicate trajectories of individually tracked microspheres. Quantitative analysis of brightfield recordings provided (E) cilia beat frequency (Hz), (F) number of epithelial cells with motile cilia per field of view, (G) cilia generated flow velocity, and (H) flow directionality. Cilia stored in 4 °C saline were isolated and hard to find, while cilia motion in these samples appeared highly variable/dyskinetic and failed to generate any meaningful microsphere movement/flow. Grey dotted lines in (G) and (H) represent values obtained from microspheres undergoing Brownian motion in an empty dish (*i.e*., no cilia-generated flow). Data is presented as Mean ± SEM (*n* ≥ 5 mice for each data point). *Significantly different from the time—matched L15+FBS value (*P* < 0.05), ^‡^ Significantly different from time 0 value for that media (*P* < 0.05). See Supplemental Material: Raw Data.xlsx for *P*-values.

The ability of the different media to maintain cilia motility following 4 °C storage was subsequently assessed (Fig. 2). Storage in saline at 4 °C caused a significant reduction in cilia motility and cilia generated flow after just 1 hour (Figs. 2A, 2E–2H), as characterized by reductions in cilia beat frequency (12.6 ± 5.2 Hz; *P* < 0.05), number of visible cells with motile cilia (4.5 ± 3.5; *P* < 0.05), cilia generated flow velocity (11.6 ± 4.6 µm/s; *P* < 0.05), and flow directionality (0.8 ± 0.23; *P* < 0.05). Except for cilia beat frequency, these parameters remained depressed or worsened as 4 °C saline storage time increased, with 12 hour samples displaying 4.5 ± 3.0 cells with motile cilia (*P* < 0.05), 6.6 ± 1.5 µm/s cilia generated flow velocity (*P* < 0.05), and 0.16 ± 0.12 flow directionality (*P* < 0.05). It should be noted that flow velocity/directionality values for 4 °C saline samples at later timepoints were generated for microspheres moving *via* Brownian motion and not due to linear cilia generated flow (dotted lines in Figs. 2G, 2H). Paradoxically, CBF increased to baseline values for 4 °C saline samples following storage for 6 hours (19.7 ± 10 Hz) or 12 hours (22.5 ± 7.7 Hz) (Fig. 2E). However, the cells with motile cilia in these groups were isolated and hard to find (had to be thoroughly searched for), while cilia motion in these samples appeared highly variable/dyskinetic and failed to generate any meaningful microsphere movement/flow (Figs. 2G, 2H).

Storage at 4 °C in the other liquid media (DPBS, NaCl/KCl, L15+FBS) resulted in no significant difference in cilia motility and cilia generated flow after 2 hour storage (Figs. 2E–2H). However, while CBF was maintained unchanged in all three media following 2 hours (DPBS: 21.2 ± 2.6 Hz, NaCl/KCl: 23.8 ± 10.4 Hz, L15+FBS: 20.3 ± 3.2 Hz), CBF was decreased at the 6 and 12 hour 4 °C timepoints for samples stored in DPBS and NaCl/KCl (Fig. 2F). A total of 4 °C storage in NaCl/KCl also resulted in a significant reduction in number of cells with motile cilia at 6 hours (11.6 ± 3.6; *P* < 0.05) and 12 hours (7.9 ± 3.6; *P* < 0.05) (Figs. 2E–2H). A reduction also seen following 4 °C storage in DPBS at 6 hours (16.1 ± 3.6; *P* < 0.05) and 12 hours (15.0 ± 4.3; *P* < 0.05). Similarly, 4 °C storage in NaCl/KCl resulted in significant reductions in cilia generated flow velocity and directionality at 6 hours (13.0 ± 4.9; *P* < 0.05) and 12 hours (12.0 ± 5.9; *P* < 0.05) for velocity, and at 12 hours (0.73 ± 0.21; *P* < 0.05) for directionality.

### Effect of storage media on cilia function following 37.5 °C storage

**Figure 3:**
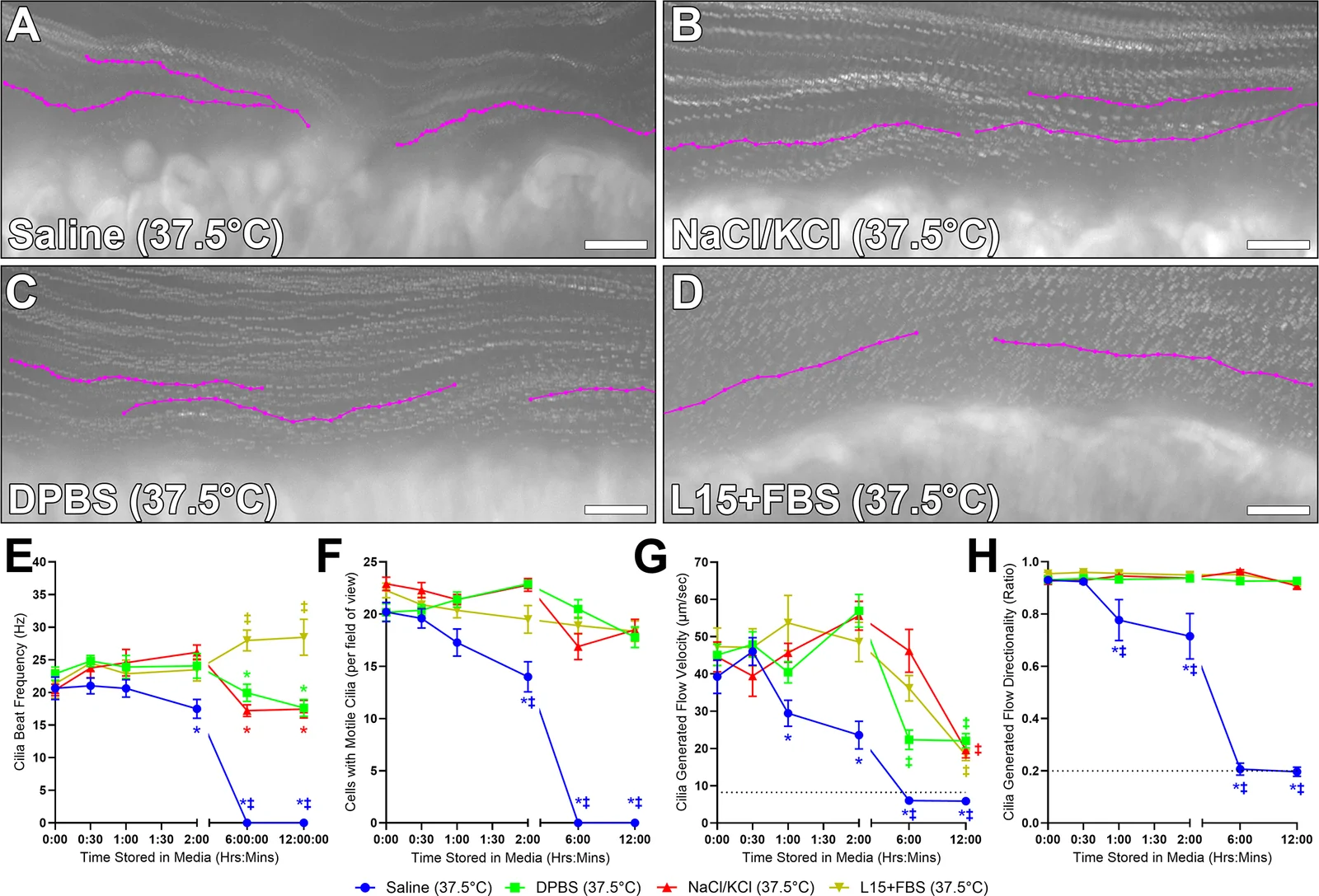
Quantification of cilia motility and cilia-generated flow in tracheal samples following storage in 37.5 °C media for 0, 30, 60, 120, 360, or 720 min. (A–D) Representative Z-projections of 10 s brightfield movies illustrating microsphere movement after 2 h of 37.5 °C storage in (A) Saline, (B) NaCl/KCl, (C) DPBS, and (D) L15+FBS. Purple traces indicate trajectories of individually tracked microspheres. Quantitative analysis of brightfield recordings provided (E) cilia beat frequency (Hz), (F) number of epithelial cells with motile cilia per field of view, (G) cilia generated flow velocity, and (H) flow directionality. Grey dotted lines in (G) and (H) represent values obtained from microspheres undergoing Brownian motion in an empty dish (*i.e*., no cilia-generated flow). Data is presented as Mean ± SEM (*n* ≥ 5 mice for each data point). *Significantly different from the time-matched L15+FBS value (*P* < 0.05), ^‡^ Significantly different to the time 0 value for that media (*P* < 0.05). See Supplemental Material: Raw Data.xlsx for *P*-values.

The ability of the different media to maintain cilia motility following 37.5 °C storage was then assessed (Fig. 3). While still worse than other media, storage in 37.5 °C saline was noticeably better at maintaining cilia motility and cilia generated flow than following storage at 4 °C (Figs. 3E–3H). One hour storage in 37.5 °C saline caused significant reductions in cilia generated flow velocity (29.4 ± 13.5 µm/sec; *P* < 0.05), and flow directionality (0.78 ± 0.3; *P* < 0.05) (Figs. 3G, 3H), and after 2 hours significant reductions in cilia beat frequency (17.5 ± 4.6 Hz; *P* < 0.05) and number of cells with motile cilia (14.0 ± 4.6; *P* < 0.05) (Figs. 3E, 3F). Longer storage times in 37.5 °C saline caused the total loss of all cilia motility, with no cells visible with motile cilia after 6 hours (Fig. 3F), thus zero CBF (Fig. 3E) and microsphere movement falling to that of Brownian motion (Figs. 3G, 3H).

**Figure 4:**
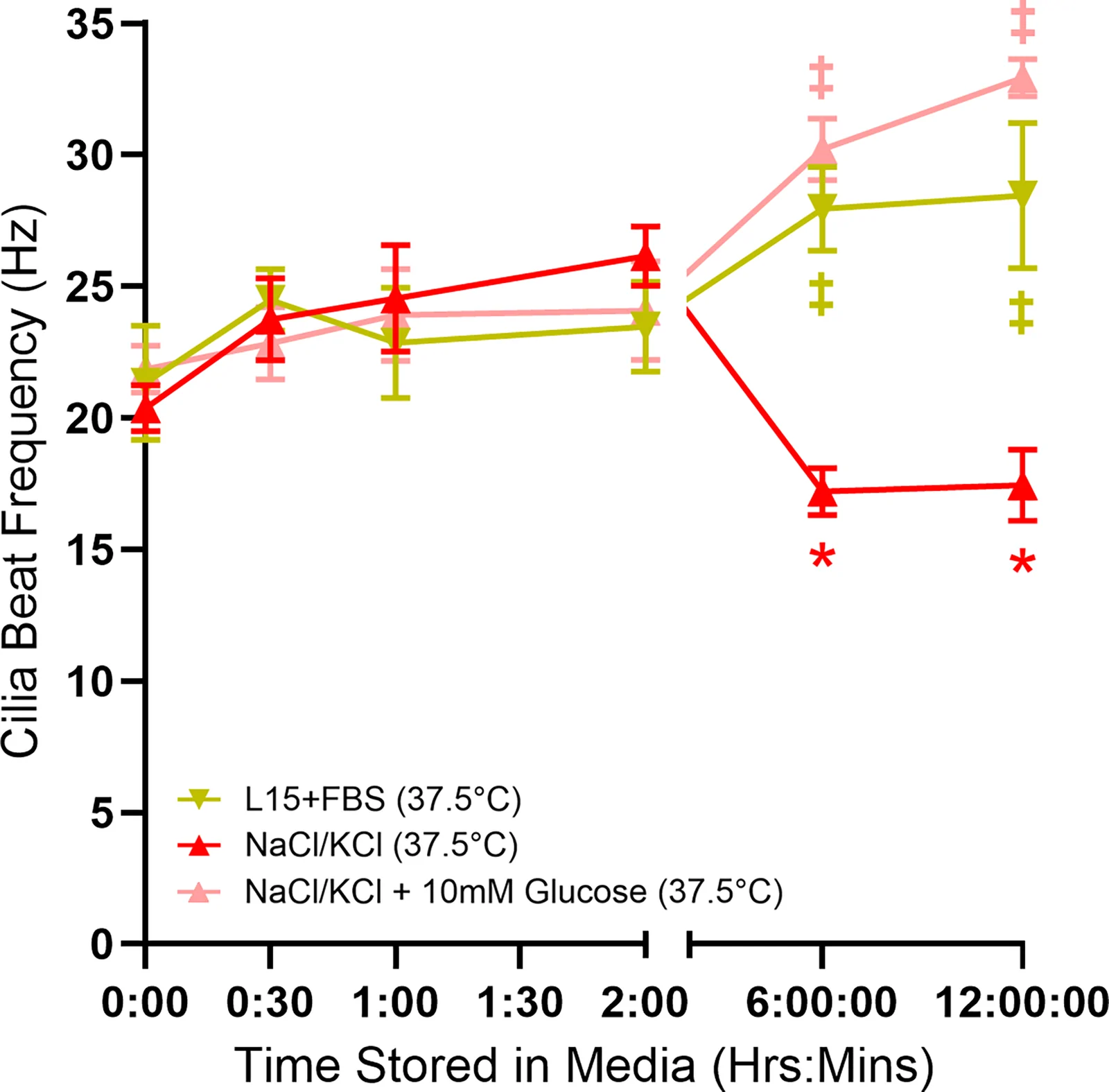
Addition of 10 mM glucose to NaCl/KCl media reverses the drop in CBF observed after 120 or 360 min storage at 37.5 °C. Data presented as Mean ± SEM (*n* ≥ 5 mice for each data point). *Significantly different from time-matched L15+FBS value (*P* < 0.05), ^‡^Significantly different to the time 0 value for that media (*P* < 0.05). See Supplemental Material: Raw Data.xlsx for *P*-values.

Storage at 37.5 °C in the other liquid media (DPBS, NaCl/KCl, L15+FBS) resulted in no significant difference in cilia motility and cilia generated flow after 2 hour storage (Figs. 3E–3H), with CBF, number of cells with motile cilia, and flow velocity/directionality all maintained at similar levels as freshly harvested/imaged tissue (Fig. 1). While the number of cells with motile cilia, and flow velocity/directionality remained similar between all three groups after 12 hour storage (Figs. 3E–3H), flow velocity was significantly reduced at 12 hours in all groups compared to the time 0 values (Fig. 3G). Of note, CBF was significantly reduced after 6 hour storage in 37.5 °C DPBS (19.9 ± 4.2 Hz; *P* < 0.05) and NaCl/KCl (17.2 ± 2.9 Hz; *P* < 0.05) compared to the L15+FBS group (28.0 ± 5.3), with no further reduction in CBF after 12 hour storage (Fig. 3E). To assess if the observed CBF reductions in simple media following 6 hour 37.5 °C storage is consistent with the lack of glucose (*i.e.*, energy source), fresh samples were incubated in NaCl/KCl+10 mM glucose for 0–12 hours and CBF was assessed (Fig. 4). It was found that addition of 10 mM glucose prevented this drop of CBF after 6 hours (30.2 ± 3.9 Hz) and 12 hours (32.9 ± 2.4 Hz), which was not significantly different to samples stored in L15+FBS after 6 hours (28.0 ± 5.3 Hz) and 12 hours (28.5 ± 8.3 Hz). It should be noted that CBF was significantly elevated (*P* < 0.05) in both NaCl/KCl+10 mM glucose and L15+FBS after 6–12 hours when compared to freshly harvested tissue (time = 0) (Fig. 4).

### Effect of storage media on tracheal epithelia cell viability

**Figure 5:**
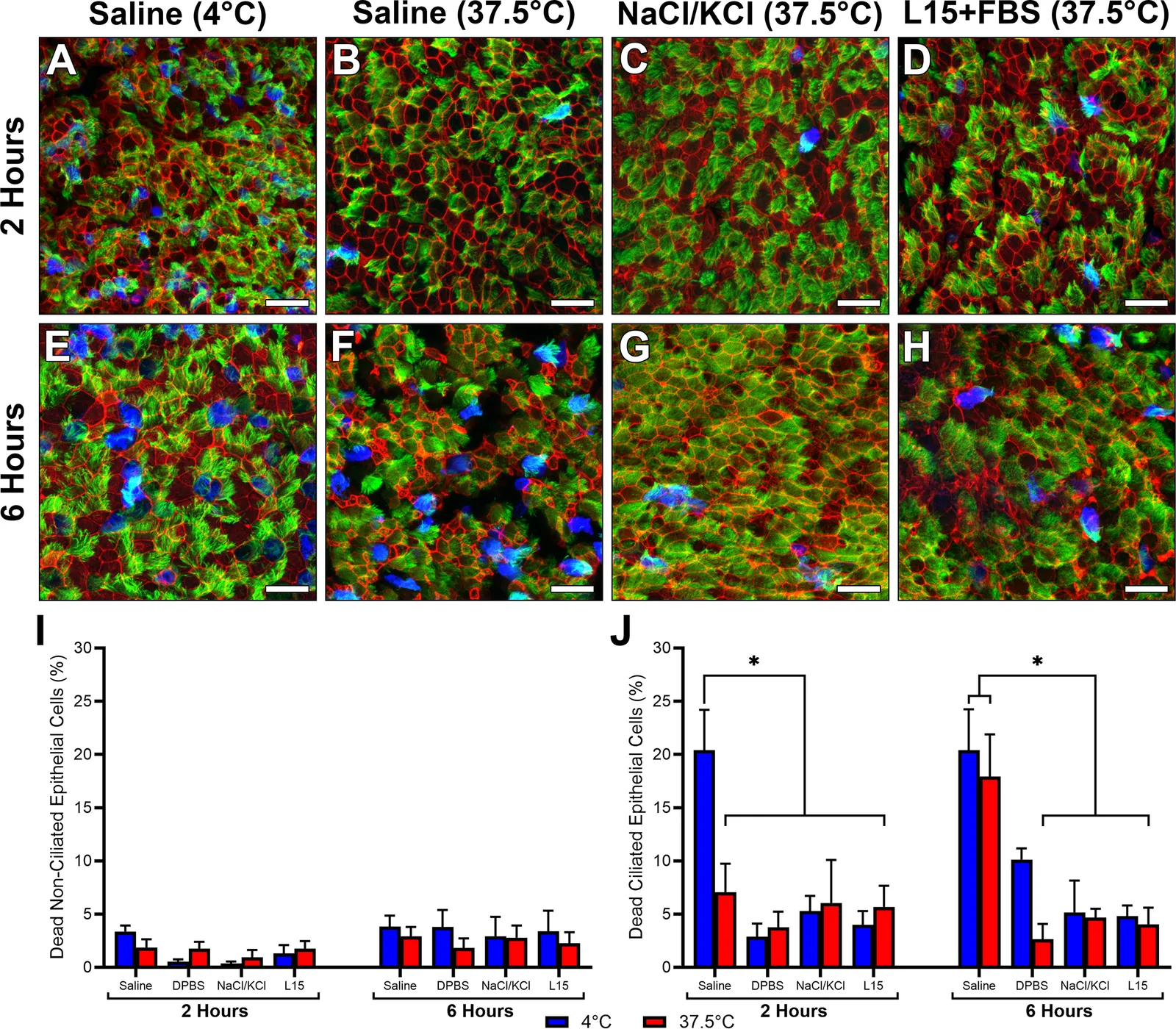
Viability of tracheal epithelial cells in tested media following storage at 4 °C or 37.5 °C for 2 or 6 hours. (A–H) Representative examples following storage in 4 °C saline (A, E), 37.5 °C saline (B, F), 37.5 °C NaCl/KCl (C, G), or 37.5 °C L15+FBS (D, H). Quantification of cell death (%) for non-ciliated (I) and ciliated (J) trachea epithelial cells following storage under the same conditions. Blue: fluorescent live/dead staining (dead cells); Green: FITC a-tubulin staining (ciliated cells); Red: Phalloidin-TRITC (cell borders). Scale bars = 20 µm. Data is presented as Mean ± SEM (*n* ≥ 5 mice for each data point). *Significant difference between indicated groups (*P* < 0.05). See Supplemental Material: Raw Data.xlsx for *P*-values.

Live/dead staining was used to assess the ability of the different media to maintain tracheal epithelia viability following 2 or 6 hour storage at either 4 °C or 37.5 °C (Fig. 5). Tracheal epithelia in all media after both 2 and 6 hours displayed a regular cobblestone arrangement with abundant numbers of ciliated epithelial cells (Figs. 5A–5H). Cell counting revealed that there was no significant difference in non-ciliated epithelia cell death for samples in all media and temperatures (Fig. 5I). However, ciliated epithelia cells appeared more sensitive to injury than their non-ciliated counterparts, with samples stored in 4 °C saline for both 2 and 6 hours showing significant elevations in ciliated epithelia cell death (Fig. 5J). A similar significant elevation in ciliated cell death was seen in samples stored in 37.5 °C saline after 4 hours (Fig. 5J). No significant difference in ciliated cell death was seen for all the other samples stored in all other media (DPBS, NaCl/KCl, L15+FBS) at either timepoint (2, 6 hours) or temperature (4 °C, 37.5 °C) (Fig. 5J).

## Discussion

The main findings of this study can be summarized as follows. Firstly, ciliary motility in mouse airway samples was preserved for up to 2 hours when tissues were stored in a simple NaCl/KCl medium formulated to approximate the ionic composition of mammalian extracellular fluid. In contrast, storage in normal saline alone led to a rapid and significant impairment of ciliary motility. Notably, this impairment was paradoxically more pronounced following storage at 4 °C than at 37.5 °C. Secondly, after 2 hours storage, ciliary motility was reduced in media lacking an exogenous energy source, irrespective of storage temperature. However, addition of 10 mM glucose was able to prevent this reduction. Finally, ciliated airway epithelial cells were more susceptible to storage associated injury and cell death than non-ciliated epithelial cells. This vulnerability was further exacerbated by storage in normal saline, which resulted in significantly increased cell death after 2 or 6 hours. Consistent with the motility findings, a temperature effect was also observed for cell death, with 2 hour storage in normal saline at 4 °C causing significantly greater ciliated cell death than storage at 37.5 °C.

### Minimum media composition requirements for cilia motility studies

Respiratory cilia function is commonly studied by measuring CBF (Bauer et al., 2024; Bishop et al., 2024; Welchering et al., 2015). However, there is currently no standardized method for collecting, storing, or imaging of these ciliated tissues, resulting in a wide range of techniques being used. This is particularly evident by the extensive variety of culture media used to maintain these ciliated tissues, including saline (Kelly et al., 2023), phosphate-buffered saline (PBS) (Mateos-Quiros et al., 2021), Hanks’ balanced salt solution (HBSS) (Chen, Lemieux & Wong, 2016; Jing et al., 2017), Medium 199 (M199) (Bauer et al., 2024; Bricmont et al., 2023; Price et al., 2015), Roswell Park Memorial Institute medium (RPMI) (Behr et al., 2023; Bishop et al., 2024), Leibovitz’s L-15 medium (L-15) (Scopulovic et al., 2022; Zahid et al., 2020), and Dulbecco’s modified eagle’s medium (DMEM) (Lever et al., 2024; Reula et al., 2021). This methodological heterogeneity prompted a previous study to determine if different media could influence cilia function, where it was found that all commonly used media displayed a similar capacity for maintaining respiratory cilia motility activity for up to 12 hours following storage at both 4 °C and room temperature (Francis, 2025a). One unexpected finding from this study was that DPBS, a simple balanced salt solution, appeared just as capable as more complex nutrient-rich media (*e.g.*, RPMI, DMEM, L15) in preserving ciliary motility in mouse airway samples (Francis, 2025a). This prompted the current study to determine the minimal media requirements necessary to maintain respiratory cilia motility *ex vivo* for cilia motility assessment.

CBF in fresh samples (time = 0) was similar to previously published values observed for both mice and humans (Delmotte & Sanderson, 2006; Yasuda et al., 2020). However, there is considerable heterogeneity within the literature for CBFs for both mouse and human airway cilia. This variability likely reflects differences in experimental setups and imaging temperatures, as well as tissue harvest techniques. In human samples, methods such as brushing or scraping can damage the ciliated epithelium, leading to reduced ciliary beat frequency and increased dyskinesia (Thomas, Rutman & O’Callaghan, 2009).

The systematic assessment of simple salt solutions in this study revealed that a media formulated to approximate the normal extracellular fluid concentration of Na^+^, Cl^−^, and K^+^ (Friedman, 2010) was sufficient to maintain cilia motile function for up to 2 hours. Beyond 2 hours the only additional requirement was glucose supplementation to prevent a small but significant reduction in CBF. These findings were independent of storage temperature, with comparable results observed at both 4 °C and 37.5 °C. The use of less complex media for short term assessment of respiratory ciliary motility in freshly harvested samples offers several possible advantages. Traditional cell culture media are designed for long term maintenance of cells/tissues and contain a plethora of ingredients (McKee & Komarova, 2017; Weiskirchen et al., 2023) that may inadvertently influence ciliary physiology. For example, previous work using the same animal model presented here demonstrated that L15 media without FBS supplementation causes an approximately 50% reduction in CBF after 30 min incubation, an effect not observed with other culture media or with L15+FBS (Francis, 2025a). While the cause of this reduction is still under investigation, this finding highlights the potential confounding effects of using culture media with complex formulations, their cocktail of differing ingredients introduces a range of exogenous factors that may unknowingly influence cilia function. The use of a simple NaCl/KCl medium (± glucose depending on incubation duration) minimizes uncontrolled variables and allows clearer interrogation of the intrinsic ciliary physiology. Another advantage that should not be overlooked is cost. With reductions in research funding (Faiman, 2025) and skyrocketing reagent costs (Woolston, 2023), using simple salt solutions to study respiratory cilia physiology, which are only a fraction of the cost of cell culture media, is an effective way to cut costs without sacrificing research quality.

### Role of storage temperature on cilia motility

Another striking finding from this study was the influence of storage temperature on the ability of different media to preserve ciliary function. Overall, samples stored at 4 °C exhibited worse ciliary motility than those stored at 37.5 °C, with a potential relationship between media complexity and resistance to hypothermic injury (Fig. 2 *vs*. Fig. 3). Storage in normal saline at 4 °C caused an early reduction in motile cilia parameters within 30 min, whereas samples stored at 37.5 °C were not affected until the 2 hour time point (Figs. 2E–2H). Similarly, storage in NaCl/KCl at 4 °C caused a small reduction in CBF after 2 hours compared to with 37.5 °C (Figs. 2E–2H), while samples stored in DPBS showed the smallest temperature dependent difference after 2 hours (Figs. 2E–2H). In contrast, samples stored in L-15 medium exhibited comparable ciliary motility across the full 12 hour study period at both temperatures. Taken together this data suggests a range of possibilities. Clearly, L15 (and other complete cell culture media) contain ingredients that protect against hypothermic injury, with this protection diminishing as media formulations become less complex. Known hypothermia protective components within culture media include: antioxidants that neutralize reactive oxygen species generated during hypothermic storage and subsequent rewarming (Alva, Palomeque & Carbonell, 2013), energy substrates such as glucose and adenosine that support cell function at reduced temperatures and during rewarming (Jakobsen et al., 2010), and simple ions and amino acids such as Ca^2+^/Mg^2+^/glutamic acid which help regulate osmotic pressure, maintain membrane potential, and suppress cell swelling (Petrat et al., 2012). However, emerging evidence suggests that 4 °C storage itself may be suboptimal for airway tissue preservation in general (Ali et al., 2021; Yamanashi et al., 2025). Improved preservation of human donor lungs has been reported at 10 °C compared with 4 °C, attributed to reduced hypothermic induced mitochondria injury (Ali et al., 2021; Yamanashi et al., 2025). Further studies are required to determine whether storage at 10 °C similarly improves preservation of ciliary motility.

The significant increase in CBF observed after 6–12 hours for samples stored in L15+FBS and NaCl/KCl+Glucose matches previous findings for samples stored in a variety of other media (HBSS, RPMI, DMEM, MEM) which was prevented by 4 °C storage (Francis, 2025a). These observations, along with the observation that it appears glucose dependent, may suggest a metabolic process is occurring that stimulates cilia activity in these samples. Exactly what is driving this elevation requires further work in future studies.

### Media composition, storage temperature, and cell death

This study further reiterates that ciliated respiratory epithelial cells are significantly more sensitive to injury and cell death than their non-ciliated counterparts (Francis, 2023; Tilley et al., 2015). Across all tested media, a consistent trend toward greater ciliated *vs*. non-ciliated cell death was observed, regardless of storage time or temperature (Fig. 5). This effect was most pronounced for samples stored in normal saline, where 2 hour storage at 4 °C resulted in cell death in approximately 20% of ciliated cells compared with less than 4% of non-ciliated cells (Figs. 5I, 5J). The impact of storage temperature was also highlighted in these samples, as ciliated cell death following 2 hour storage in normal saline at 37.5 °C was not significantly elevated and only increased after 6 hours of storage (Fig. 5J). A major challenge in studying ciliated airway epithelial cells is this sensitivity to injury, which can be caused during any step of the experimental process, such as tissue collection, temperature changes, and *ex vivo* storage conditions which can all rapidly disrupt epithelial integrity, impair ciliary motility, and induce selective injury/death of these ciliated cells (Thomas, Rutman & O’Callaghan, 2009; Tilley et al., 2015). This intrinsic fragility complicates experimental reproducibility and interpretation, underscoring the need for standardized, cilia preserving protocols for studying respiratory cilia motility. A loss of integrity of these ciliated cells under stress conditions may occur early in the cascade of events leading to chronic airway inflammatory diseases in humans such as asthma and COPD which display impaired ciliogenesis, ciliary beating and ultrastructural defects (Guan et al., 2018).

### Study limitations

As this study was conducted using animal samples further work will be necessary to confirm the findings of this study with human samples, especially for possible standardization of handling protocols for potential diagnostic purposes. However, as animal models are commonly used to study cilia motility (Bustamante-Marin & Ostrowski, 2017; Lyu et al., 2024) this data should prove useful for future studies assessing motile cilia function.

Another possible limitation of this study is its limited size, which reflects its design more as a pilot study. The primary objective was to evaluate the feasibility of using simple NaCl/KCl solutions as economical alternatives to established culture media (such as L15) for assessing ciliary motility in mouse respiratory preparations. These preliminary findings will require validation through larger-scale studies before broader application can be recommended.

## Conclusions

In conclusion, this study demonstrates that complex and expensive cell culture media is not required for short-term assessment of respiratory ciliary motility in freshly harvested airway samples. A simple media approximating physiological extracellular concentrations of Na^+^, Cl^−^, and K^+^ was sufficient to preserve ciliary motility for up to 2 hours at 37.5 °C, with only glucose supplementation required only beyond this time point to prevent a modest reduction in CBF. However, prolonged storage at 4 °C (2–12 hours) was associated with reductions in ciliary motility and should be avoided when possible. The use of a simple media to conduct cilia motility studies will help minimize exogenous factors that could influence cilia function, enabling clearer insights into the underlying pathways regulating motile cilia function. Moreover, identifying the most basic solution capable of maintaining motility will provide a standardized and cost-effective foundation for future mechanistic studies.

## Supplemental Information

10.7717/peerj.21674/supp-1 Supplemental Information 1 Raw Data and P-Values.

10.7717/peerj.21674/supp-2 Supplemental Information 2 List of all experimental groups and treatments used in study. All animals and samples collected from each animal are listed including media used and temperature/storage times tested.

10.7717/peerj.21674/supp-3 Supplemental Information 3 ARRIVE Checklist.

## Additional Information and Declarations

### Data Availability

The following information was supplied regarding data availability:

The data is available in the Supplemental Files.
