# Supplementary material for: Defining minimum media requirements for ex vivo respiratory cilia studies using mouse tracheal tissue
Source: PeerJ. 2026 Sep 7;14:e21674. doi: 10.7717/peerj.21674 (PMC13564408; doi:10.7717/peerj.21674)
Supplement: Supplementary file 2 [file peerj-14-21674-s002.docx]

| **Experiment** | **Media** | **Storage Times** | **Storage Temperature** | **Animal Number** | **Samples  per Animal^†^** | **Total Samples** |
| --- | --- | --- | --- | --- | --- | --- |
| VideoMicroscopy | Saline | 0, 30, 60, 120 mins | 4°C | 5 | 4 | 20 |
| VideoMicroscopy | Saline | 360, 720 mins | 4°C | 5 | 2 | 10 |
| VideoMicroscopy | Saline | 0, 30, 60, 120 mins | 37°C | 5 | 4 | 20 |
| VideoMicroscopy | Saline | 360, 720 mins | 37°C | 5 | 2 | 10 |
| VideoMicroscopy | DPBS | 0, 30, 60, 120 mins | 4°C | 5 | 4 | 20 |
| VideoMicroscopy | DPBS | 360, 720 mins | 4°C | 5 | 2 | 10 |
| VideoMicroscopy | DPBS | 0, 30, 60, 120 mins | 37°C | 5 | 4 | 20 |
| VideoMicroscopy | DPBS | 360, 720 mins | 37°C | 5 | 2 | 10 |
| VideoMicroscopy | NaCl/KCl | 0, 30, 60, 120 mins | 4°C | 5 | 4 | 20 |
| VideoMicroscopy | NaCl/KCl | 360, 720 mins | 4°C | 5 | 2 | 10 |
| VideoMicroscopy | NaCl/KCl | 0, 30, 60, 120 mins | 37°C | 5 | 4 | 20 |
| VideoMicroscopy | NaCl/KCl | 360, 720 mins | 37°C | 5 | 2 | 10 |
| VideoMicroscopy | NaCl/KCl  + 10mM Glucose | 0, 30, 60, 120 mins | 37°C | 5 | 4 | 20 |
| VideoMicroscopy | NaCl/KCl  + 10mM Glucose | 360, 720 mins | 37°C | 5 | 2 | 10 |
| VideoMicroscopy | L15+FBS | 0, 30, 60, 120 mins | 4°C | 5 | 4 | 20 |
| VideoMicroscopy | L15+FBS | 360, 720 mins | 4°C | 5 | 2 | 10 |
| VideoMicroscopy | L15+FBS | 0, 30, 60, 120 mins | 37°C | 5 | 4 | 20 |
| VideoMicroscopy | L15+FBS | 360, 720 mins | 37°C | 5 | 2 | 10 |
| Live/Dead Staining | Saline, DPBS, NaCl/KCl, L15+FBS | 120 mins | 4°C or 37°C | 5 | 8 | 40 |
| Live/Dead Staining | Saline, DPBS, NaCl/KCl, L15+FBS | 360 mins | 4°C or 37°C | 5 | 8 | 40 |
| ^†^One sample per animal per storage time | | | **Total Animals:** | 100 | **Total Samples:** | 350 |
